# Acute Myeloid Leukemia With Philadelphia Chromosome and Complex Karyotype: A Diagnostic Dilemma

**DOI:** 10.7759/cureus.109808

**Published:** 2026-05-28

**Authors:** Sandip Shah, Shabiha Modan, Parloop A Bhatt

**Affiliations:** 1 Department of Hematology and Medical Oncology, Hemato Oncology Clinic Vedanta, Ahmedabad, IND; 2 Department of Pharmacy, Gujarat Technological University, Ahmedabad, IND; 3 Department of Research, Marengo Care Institute of Medical Sciences (CIMS) Hospital, Ahmedabad, IND

**Keywords:** acute myeloid leukemia (aml), complex karyotype, imatinib treatment, philadelphia chromosome-positive (ph+), tetrasomies, trisomies, tyrosine kinase inhibitors (tki)

## Abstract

Acute myeloid leukemia (AML) is a hematological malignancy driven by the clonal expansion of immature myeloid cells, with cytogenetic findings playing a defining role in both its diagnosis and risk stratification. The Philadelphia chromosome, generated by the t(9;22)(q34;q11.2) translocation and the resulting BCR-ABL1 fusion gene, is a well-known driver of chronic myeloid leukemia (CML). Its occurrence in AML, however, is uncommon and carries distinct diagnostic and therapeutic implications that are not always straightforward to navigate.

We report a 47-year-old male patient who came to us with a 10-day history of fever, hip pain, and back pain. Blood tests showed anemia and thrombocytopenia, with 49% blasts on peripheral smear. Bone marrow biopsy confirmed acute leukemia with 63% blasts. Cytogenetic studies revealed t(9;22) along with a remarkably complex karyotype, with trisomies of eight chromosomes and tetrasomies of three others. The patient received high-dose cytarabine over three cycles, developed febrile neutropenia requiring amikacin, and was subsequently started on imatinib 400 mg in view of the BCR-ABL1 positivity. He has remained clinically stable at six months of follow-up.

To our knowledge, finding Philadelphia chromosome-positive acute myeloid leukemia (Ph+ AML) alongside such a degree of additional chromosomal numerical gains is exceptionally rare. What makes this case worth reporting is the convergence of three issues that each present their own challenges: establishing a diagnosis of de novo AML rather than CML in blast crisis, managing an already aggressive disease made more so by a complex karyotype, and deciding on the role of tyrosine kinase inhibitors in a disease setting where their use is not yet standardized. Detailed cytogenetic workup proved critical in navigating all three.

## Introduction

Acute myeloid leukemia (AML) arises from the uncontrolled proliferation of myeloid progenitor cells that have lost their ability to differentiate normally. Cytogenetic and molecular findings at diagnosis remain among the most powerful determinants of prognosis and guide how treatment is planned [1,2]. Within the wide spectrum of chromosomal abnormalities seen in AML, the Philadelphia chromosome holds a somewhat unusual place. Formed by the t(9;22)(q34;q11.2) translocation and the BCR-ABL1 fusion gene it produces, the Philadelphia chromosome is almost synonymous with chronic myeloid leukemia (CML) and is also a well-recognized feature of acute lymphoblastic leukemia. In AML, however, it is genuinely rare, seen in only around 0.3% to 3# of cases [3,4].

When a patient presents with acute leukemia and is found to carry the Philadelphia chromosome, the first and most pressing question is whether this represents true de novo AML or a blast crisis of an underlying CML that had gone undiagnosed. This distinction is not always easy to make. The morphological and cytogenetic overlap between the two can be considerable, and yet arriving at the correct answer matters enormously since the treatment pathways diverge significantly [4].

An additional layer of complexity arises when a complex karyotype is also present. By definition, a complex karyotype involves three or more unrelated chromosomal abnormalities. In AML, this finding is associated with genomic instability, a higher likelihood of TP53 mutations, poor response to standard induction therapy, and worse overall outcomes [5,6]. When t(9;22) occurs alongside such a karyotype, the clinical picture becomes even more difficult to interpret and manage.

Over the past decade, there has been growing interest in incorporating tyrosine kinase inhibitors (TKIs) such as imatinib, dasatinib, and ponatinib into the treatment of Philadelphia chromosome-positive acute myeloid leukemia (Ph+ AML), given the central oncogenic role of BCR-ABL1 kinase activity. The rationale is sound, but the evidence base is still largely built on case reports and small series rather than prospective trials, and no consensus treatment protocol currently exists [6,7].

We present this case of de novo AML with t(9;22) and a highly complex karyotype to contribute to this limited literature, describe the diagnostic reasoning that guided our approach, and discuss the treatment decisions made. This case report was prepared in accordance with the CAse REport (CARE) reporting guidelines.

## Case presentation

A 47-year-old male patient was brought to our attention with a 10-day history of fever, progressive hip pain, and lower back pain. He had no prior hematological diagnosis, had not received chemotherapy or radiation in the past, and had no family history of leukemia. On examination, he looked unwell and was visibly pale, though there was no detectable hepatosplenomegaly or lymphadenopathy.

His initial laboratory investigations are summarized in Table 1. Of note, his hemoglobin was 9.4 g/dL, total leukocyte count 5,400/cmm, and platelet count 75,000/cmm. The peripheral blood smear showed 49% blasts. Serum lactate dehydrogenase was markedly elevated at 2,570 U/L against a reference range of 140-280 U/L, pointing to a high tumor burden. Renal and hepatic parameters were within acceptable limits.

**Table 1:** Laboratory investigations at presentation

Parameter	Patient Value	Reference Range
Hemoglobin (g/dL)	9.4	Male: 13–17, Female: 12–15
RBC (million/cmm)	3.18	Male: 4.5–5.5, Female: 4.2–5.5
WBC (/cmm)	5,400	4,000–11,000
Platelets (/cmm)	75,000	150,000–400,000
Polymorphs (%)	84	40–80
Lymphocytes (%)	14	20–40
Eosinophils (%)	0	1–6
Monocytes (%)	2	2–10
Basophils (%)	0	0–1
Peripheral Blood Blasts (%)	49	0
Bone Marrow Blasts (%)	63	< 5
Serum Glutamic Pyruvic Transaminase (SGPT)/Alanine Aminotransferase (ALT; U/L)	42	7–56
Serum Creatinine (mg/dL)	1.27	0.7–1.3
Lactate Dehydrogenase (LDH; U/L)	2,570	140–280

Bone marrow aspiration and trephine biopsy were performed, and confirmed acute leukemia, with 63% myeloid blasts on examination.

**Figure 1:**
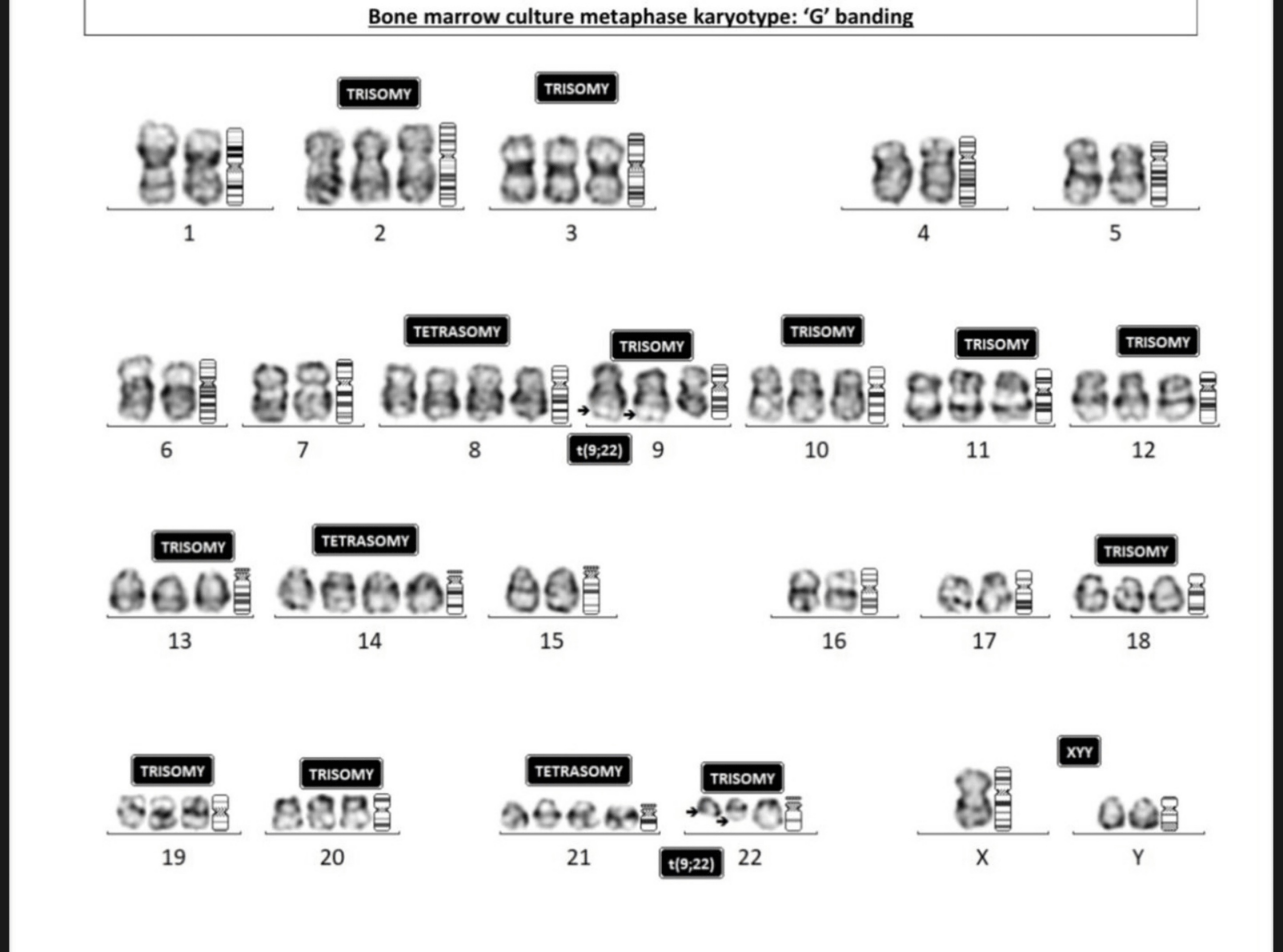
Bone marrow culture metaphase karyotype (G-banding) demonstrating the Philadelphia chromosome t(9;22) with extensive additional numerical abnormalities, including trisomies of chromosomes 2, 3, 9, 10, 11, 12, 13, 18, 19, 20, and 22; tetrasomies of chromosomes 8, 14, and 21; and XYY sex chromosome pattern.

Cytogenetic analysis using conventional G-banding returned a striking result, as illustrated in Figure 1. In addition to t(9;22)(q34;q11.2) confirming the Philadelphia chromosome, the karyotype showed trisomies of chromosomes 2, 3, 9, 10, 11, 12, 13, 18, 19, 20, and 22, tetrasomies of chromosomes 8, 14, and 21, and an XYY sex chromosome pattern. The extent of these additional numerical gains, on top of the Philadelphia translocation, was unusual and pointed to a high degree of underlying genomic instability.

Taking the clinical picture as a whole, including the absence of any prior CML history, no splenomegaly, and the cytogenetic findings, a diagnosis of de novo AML with Philadelphia chromosome and complex karyotype was made. Immunophenotyping supported a myeloid blast immunophenotype. RT-PCR for BCR-ABL1 confirmed positivity, consistent with the t(9;22) seen on karyotyping.

Diagnosis and management

Given the presence of t(9;22), it was necessary to carefully consider whether this was truly de novo AML or a presentation of CML in myeloid blast crisis. Several features argued against the blast crisis. There was no prior history of CML, no preceding constitutional symptoms, no basophilia, no splenomegaly, and the patient had never received a TKI. Blast phase CML typically declares itself against a backdrop of known CML or carries additional CML-associated cytogenetic features such as isochromosome 17q or duplication of the Philadelphia chromosome. None of these applied to our patient. On the balance of clinical and cytogenetic evidence, de novo AML was the more appropriate diagnosis.

The patient was started on high-dose cytarabine at 5 g and completed three induction cycles. His course was complicated by febrile neutropenia, during which amikacin was given, and he recovered well enough to continue treatment. Given the confirmed t(9;22) and the established role of BCR-ABL1 kinase signaling in driving disease biology, imatinib 400 mg once daily was added as targeted therapy alongside the chemotherapy backbone.

He tolerated the combined approach reasonably well. At his six-month follow-up, he remains stable, is continuing imatinib, and is undergoing repeat bone marrow assessment and molecular monitoring of BCR-ABL1 transcript levels to gauge depth of response.

## Discussion

This case is unusual for the combination of three features that each present their own challenges: the presence of the Philadelphia chromosome in AML, a karyotype far more complex than t(9;22) alone, and the diagnostic difficulty of confidently ruling out CML blast crisis. Any one of these would warrant attention on its own. Together, they made this a particularly demanding case to work through.

Philadelphia chromosome positivity in AML is genuinely rare. Registry data put it at between 0.3% and 3% of all AML cases [3,4]. A 2024 analysis of over 5,800 patients in the DATAML registry found only 20 cases of de novo BCR::ABL1-positive AML, translating to an incidence of just 0.3% [8]. These numbers underline how infrequently this cytogenetic combination is encountered in everyday clinical practice.

The question of distinguishing de novo Ph+ AML from CML in myeloid blast crisis is one that cannot be glossed over. Both conditions can present with acute leukemia and Philadelphia chromosome positivity, and the consequences of misdiagnosis are significant. The distinguishing features we relied on in this case included the absence of any prior CML history, no preceding chronic phase symptoms such as weight loss or progressive leukocytosis, no basophilia, and no splenomegaly [4]. Although our patient's karyotype was complex enough to include trisomy of chromosome 9, which can occasionally be seen in CML blast crisis, the absence of other characteristic CML evolution markers such as isochromosome 17q or Philadelphia chromosome duplication supported our conclusion of de novo AML. In genuinely ambiguous cases, ABL1 kinase domain mutation analysis may offer additional clarification [4].

The complexity of the karyotype in this case deserves particular comment. A complex karyotype, defined by three or more unrelated cytogenetic abnormalities, is already an adverse prognostic marker in AML, associated with chemotherapy resistance and poor survival [5,6]. Our patient had eight trisomies and three tetrasomies on top of t(9;22). Even within the rare subset of Ph+ AML, this degree of additional chromosomal gain is unusual. The Data from the Acute Myeloid Leukemia (DATAML) Registry Study, for example, found that when Ph+ AML patients had additional abnormalities, the t(9;22) was typically the dominant single structural change rather than one element within a massively disrupted karyotype [8]. Our case sits at an extreme end of this spectrum.

On the treatment side, the use of imatinib in Ph+ AML is supported by a growing, though still limited, body of evidence. Earlier case reports have shown that while imatinib alone is unlikely to produce or maintain complete remission in AML, it can contribute meaningfully as part of a combined strategy, particularly in sustaining molecular response after induction [9]. More recent reports have also explored combining TKIs with hypomethylating agents in patients who are not candidates for intensive chemotherapy, with some achieving molecular complete remission despite high-risk disease features [10]. The DATAML Registry, representing the largest systematic analysis of this patient group to date, showed that intensive chemotherapy combined with imatinib produced complete remission rates and relapse-free survival figures that challenge the current adverse-risk labelling of Ph+ AML under the 2022 European LeukemiaNet (ELN) guidelines [8]. Whether these outcomes extend to patients with the additional karyotypic complexity seen in our patient is less clear. Prognosis in such cases is likely worse, and durability of response is harder to achieve [6]. Second- and third-generation TKIs, including dasatinib and ponatinib, remain options for patients who do not respond adequately to imatinib or who develop ABL1 kinase domain mutations [7]. Our patient started imatinib 400 mg daily alongside cytarabine and remains well at six months, which is an encouraging start, though long-term follow-up will be needed before drawing any conclusions.

What this case reinforces, above all, is that full cytogenetic and molecular characterization at diagnosis is not optional. Identifying t(9;22) in this patient changed the treatment strategy in a fundamental way. Similar cases published in the literature have highlighted how aggressive Ph+ AML can be, particularly when atypical BCR-ABL1 transcripts or additional cytogenetic changes are present [11]. The long-established prognostic value of pretreatment karyotyping in AML [12] is only reinforced by cases like this one, where the chromosomal picture extended far beyond what was initially anticipated.

## Conclusions

We describe an unusual and clinically challenging case of de novo AML characterized by the simultaneous presence of the Philadelphia chromosome and an extensively complex karyotype involving multiple trisomies and tetrasomies. This combination represents a rare and aggressive cytogenetic profile that sits at the intersection of three clinically important themes: the rarity of Ph+ AML itself; the adverse biological significance of complex karyotypes; and the diagnostic difficulty in confidently excluding blast crisis of CML.

Through careful integration of clinical history, morphology, immunophenotyping, and cytogenetics, a diagnosis of de novo AML was established, and combined treatment with high-dose cytarabine and imatinib was initiated. The patient’s stable status at six months is an encouraging early signal, though the long-term outlook in this highly complex cytogenetic setting remains uncertain and warrants close monitoring. This case adds to the growing but still limited body of literature on Ph+ AML and underscores that comprehensive cytogenetic evaluation is not merely an academic exercise; it is a clinical imperative that directly shapes the diagnostic conclusion, risk stratification, and treatment approach in acute leukemia.
